# Multi-epitope vaccine design against *Clostridioides difficile* using the ABC-type transport system protein

**DOI:** 10.6026/973206300210990

**Published:** 2025-05-31

**Authors:** Abirami T.S., Prasanthi Saravana, Shalini U., Prasanth G., Swathi S., Abhinand Ponneri Adhithavarman

**Affiliations:** 1 Department of Bioinformatics, Sri Ramachandra Faculty of Engineering and Technology, Sri Ramachandra Institute of Higher Education and Research, Chennai - 6000 116, Tamil Nadu, India

**Keywords:** *C. difficile*, epitope, antigen, antibody, docking, immune simulation, SGD3, good health and well-being

## Abstract

*Clostridioides difficile* is a major cause of antibiotic-associated nosocomial diarrhoea with increasing global incidence and nearly one million annual cases reported in India. Therefore, it is of interest to design a multi-epitope vaccine design against *Clostridioides* difficile the ABC-type transport system protein. Hence, B-cell, CTL and HTL epitopes were predicted using BepiPred-2.0, NetCTL 1.2 and NetMHCIIpan 4.0. Thus, a 66-amino-acid epitope construct with AAY and GPGPG spacers for the TLR3 adjuvant to enhance immunogenicity was reported for further consideration. The construct was validated for antigenicity, non-allergenicity and physicochemical stability. Structural modelling, molecular docking with TLR3 (PDB ID: 2A0Z) and 100-ns molecular dynamics simulations showed excellent structural stability (RMSD ~0.1 Å) and receptor binding affinity, while immune simulations via C-ImmSim indicated strong B- and T-cell responses with elevated IFN-γ and IL-2 levels, supporting its potential as a safe, effective, and targeted prophylactic against *C. difficile* infections.

## Background:

*Clostridioides difficile* (*C. difficile*) is a bacterium that naturally resides in the intestines. It is a gram-positive, spore-forming, motile and anaerobic microorganism known for causing severe infections in the colon (large intestine). It is recognized as the primary cause of antibiotic-associated nosocomial diarrhoea [1]. It was later found that this microorganism was capable of producing toxins. In 2016, it was reclassified from the *Clostridium* genus to Clostridioides due to taxonomic differences. Despite the name change, it remains commonly referred to as *C. diff* [2]. While *C. difficile* is primarily associated with hospital-acquired infections, about one-third of cases originate from hospital settings due to transmission from infected individuals [3, 4]. Only a few antibiotics have been directly linked to *C. difficile* infections (CDI). The bacterium is naturally present in the gut but remains harmless unless the gut's beneficial bacteria are eliminated due to prolonged antibiotic use. Antibiotics disrupt the balance of gut microbiota, allowing *C. difficile* to proliferate, degrade the mucus lining of the colon and trigger colitis an inflammatory reaction in the intestines. *Clostridioides difficile* produces two major toxins-enterotoxin A and cytotoxin B which cause significant damage by destroying healthy cells in the colon. While the bacterium is relatively inactive outside the human body and has reduced motility, it can survive for extended periods on surfaces, making transmission relatively easy. The spores can remain dormant for years and reactivate upon re-entering the gut, leading to infection [5]. The incidence of *Clostridioides difficile* infection and its associated mortality rate have increased significantly in recent years [6]. The bacteria can spread through direct contact or ingestion of contaminated food and water. The severity of infection varies from mild discomfort to life-threatening conditions. *Clostridioides difficile* infection symptoms include abdominal pain, watery diarrhoea (seen in 60% of cases), fever and nausea [7]. In rare cases, *C. difficile* can perforate the intestines, leading to sepsis-a life-threatening immune response to infection. Some individuals carry *C. difficile* without experiencing symptoms, acting as carriers who can unknowingly transmit the infection to others.

*C. difficile* is responsible for approximately 20% of all antibiotic-associated diarrhoea cases and is widespread in India, with nearly one million diagnoses annually. Key risk factors for *Clostridioides difficile* infection include male gender, age over 65 or under 1 year (with existing health conditions), prolonged hospital stays, immunodeficiency disorders (e.g., HIV), malnutrition, inflammatory bowel diseases (IBDs), diabetes, cystic fibrosis and low serum albumin levels (<2.5 g/dL). *Clostridioides difficile* infection spores can persist even in the presence of antibiotics like metronidazole and vancomycin, which are used for treatment [8, 9]. Diagnostic limitations in India often lead to missed cases of CDI. The pathogenesis of *Clostridioides difficile* infection remains complex, as the relationship between *C. difficile* colonization and infection is not entirely understood. Clinically, *Clostridioides difficile* infection is diagnosed based on symptoms, particularly diarrhoea following antibiotic use. Though metronidazole and vancomycin are commonly used treatments, some *C. difficile* strains have developed resistance. Anti-diarrheal drugs are generally avoided as they can worsen colitis. For recurrent *Clostridioides difficile* infection cases, fidaxomicin, a novel macrocyclic antibiotic, is recommended over oral vancomycin. Faecal microbiota transplantation (FMT) is another therapeutic option, but its complexity and variability present challenges. Further research is needed to explore bacterial-epithelial interactions and develop vaccines that prevent colonization and infection [10]. Development of a vaccine against centers singularly or multiply at fault for adherence, colonization and persistence is much favorable to a multifaceted one developing vaccines. An antibody attaches to a particular small area of an antigen known as the epitope. An epitope denotes one area that an antibody can bind, therefore, a single antigen can have several epitopes. Immune-informatics is being applied to bring forward knowledge-aided design of multi-epitope vaccines. This approach enhances accuracy in targeting and formulating vaccines that can easily change as they incorporate short peptide fragments [11, 12, 13-14]. Distinction between multi epitope vaccines and other classical vaccines, has a different design concept, less expensive to create, the efficacy can overcome wet lab activities, time saving, more stable [15]. The multi-epitope vaccine designs do not encompass the whole pathogen hence they pose little risks; they are also very selective [16]. The *C. difficile* vaccines are in the pre-clinical stages, but the product also holds potential options for the prevention and treatment of the CDI. There is several evidence such a vaccine could treat the infections [17]. An immuno-informatics approach toward the introduction of a novel multi-epitope vaccine against *Clostridium* difficile is available [24-25]. Therefore, it is of interest to develop short peptide vaccine candidates to combat *C. difficile* infection using the ABC-type transport system protein.

## Methodology:

## Selection of proteins for vaccine development:

A subtractive proteomic approach was employed to identify a suitable target protein for vaccine design against *Clostridioides difficile*. Protein pathways common to both *C. difficile* and Homo sapiens were first identified using the KEGG database, allowing the exclusion of host-conserved proteins to minimize potential cross-reactivity. Unique proteins specific to *C. difficile* were then analyzed, and their full amino acid sequences were retrieved from the NCBI database in FASTA format. To prioritize virulent and antigenic targets, the VirulentPred server was used to assess the virulence potential of these proteins. Based on this systematic screening, a highly immunogenic and pathogen-specific protein was selected as the optimal candidate for multi-epitope vaccine design.

## Prediction of linear B-cell epitopes:

Linear B-cell epitopes were predicted using BepiPred-2.0, a robust tool employing a random forest algorithm trained on structurally defined epitopes from antibody-antigen complexes. This approach outperforms earlier methods like LBtopeand BLAST-based predictors in accuracy (AUC: 0.65 vs. 0.58-0.62). The longest predicted epitope (LTFKKR) was prioritized for its antigenic potential. Comparative analyses with contemporary methods such as LBCE-BERT, which uses XGBoost classifiers on BERT embeddings, highlight BepiPred-2.0's balance of specificity (81.6%) and sensitivity (63.5%) [18].

## Cytotoxic T-lymphocyte epitope prediction:

Cytotoxic T epitopes were identified via Net cytotoxic T 1.2 [25]; integrating MHC-I binding, proteasomal cleavage, and TAP transport efficiency. A threshold of 0.75 ensured high specificity (>80%) while retaining sensitivity for rare alleles5. Epitopes with IC50 values <0.4 nM were selected, aligning with benchmarks demonstrating strong immunogenicity correlations.

## Helper T-Lymphocyte (HTL) epitope prediction:

Helper T-lymphocyte epitopes were predicted using NetMHCIIpan 4.0, which leverages artificial neural networks for pan-specific MHC-II binding affinities. Epitopes with binding scores ≤2.0 (strong binders) and antigenicity thresholds <0.4 were retained, consistent with criteria optimizing CD4+ T-cell activation [19].

## Design of a multi-epitope vaccine candidate:

The predicted linear B-cell epitopes, high-affinity helper T-lymphocyte epitopes, and strong cytotoxic T lymphocytes epitopes were integrated to construct a multi-epitope vaccine. The vaccine was designed to be both antigenic and non-allergenic by incorporating chimeric sequences containing helper T-lymphocyte and cytotoxic T lymphocytes epitopes. The final vaccine construct included two cytotoxic T lymphocytes epitopes, one helper T-lymphocyte epitope, and four INF-γ epitopes per selected protein. These epitopes were linked using GPGPG linkers to minimize junctional epitope formation and enhance immune recognition. Additionally, AAY linkers were utilized to optimize the sequence further. To enhance immunogenicity, the Toll-like receptor-3 (TLR 3) molecular adjuvant was selected as a target. TLR3 recognizes viral double-stranded RNA and plays a key role in initiating immune responses. The sequence for TLR3_HUMAN was obtained from UniProtKB (Uniprot ID: O15455) [20]. It was fused at the N-terminal with an EAAAK linker and at the C-terminal with eight histidine residues to facilitate purification [4]. The final vaccine construct, consisting of 66 amino acids, was successfully developed. The schematic representation of the designed vaccine along with the linker sequences is depicted in Figure 2 - (see PDF).

The resigned vaccine sequence is as follows:

N'EAAAKLTFKKRKKAVDNISLAIIISNGVMGYAAYSIYGLLGPNGAGKSTYGLLGPNGAGPGPGTAT-C'

## IFN-γ epitopes prediction -gamma inducing epitopes:

To incorporate IFN-γ stimulation into the vaccine design, epitopes were predicted from all the three protein types: B-cell, cytotoxic T and helper T-lymphocyte. The best scoring epitopes were chosen from each protein for the construction of the final vaccine. The potential epitopes were analyzed using the IFN-γ epitope server. The prediction was done on a Support Vector Machine model, trained to differentiate between IFN-γ and non- IFN-γ epitopes. By analysing the overlapping sequences obtained from the submitted proteins, the server predicts the presence of IFN-γ epitopes.

## Antigenicity, allergenicity and physicochemical property prediction:

The candidate's Antigenicity was predicted using ANTIGENpro and its Allergenicity predicted using AllerTOP v2.0. Designed sequences for the vaccine candidate were uploaded to the ANTIGENpro server as plain sequence and additionally email ID was given to get the antigenicity score in mail. The various physiochemical properties were analysed using Prot Param.

## Secondary structure prediction:

To predict - secondary structure option PSIPRED Server was used. An E-mail id was provided to facilitate the request. Secondary structure analysis for the candidate sequence took place and, in a minute, or two the result was mailed back.

## Tertiary structure modeling:

The Ab-initio modeling of the multi-epitope protein was done using Robetta web server. Here the vaccine sequence was given as input in FASTA format and selected Ab-initio only to model the structure of the vaccine. The 3D model obtained was fine-tuned using the ModRefiner from the Zhanglab server and this refined model was then validated using PDBsum Ramachandran plot analysis. It took 5 hours to get the refined modeled structure.

## Molecular docking of designed protein with TLR3:

## Protein peptide docking:

Toll-like receptors (TLRs) are single-pass transmembrane receptors that play a crucial role in the innate immune response [20]. The interaction between TLR3 and the peptide was predicted using a hybrid template-based docking algorithm. Molecular docking studies were performed using the HDock protein-protein docking server (Yan *et al.* 2020), with TLR3 (PDB ID: 2A0Z) as the target. The server employed a hybrid approach integrating template-based docking to enhance interaction predictions. From the generated models, the top 10 best-ranked structures were analyzed, and the model with the lowest binding energy was selected for further evaluation.

## MD simulation:

The molecular dynamics (MD) simulations were conducted using Desmond 2020.1 (Schrödinger, LLC) to analyze TL3-peptide complexes in SPC water, employing the OPLS-2005 force field. The simulations were carried out within a 10 Å x 10 Å x 10 Å periodic boundary box, with Na^+^ ions ensuring charge neutrality and NaCl maintaining physiological conditions. System equilibration involved a 100 ns NVT ensemble, followed by a 50 ns NPT ensemble at 27°C and 1 bar pressure. A 2-fs time step was used, with a 100 ps relaxation phase controlled by the Martyna-Tuckerman-Klein barostat. Long-range electrostatic interactions were computed using the particle mesh Ewald method with a 9 nm cutoff. Bonded forces were calculated via the RESPA integrator. Simulation stability was assessed based on parameters such as RMSD, RMSF, and radius of gyration, with frames analyzed every 25 ns. The results indicate that the docked complex remained stable throughout the simulation, with minimal structural deviations, supporting the robustness of the interaction and the structural integrity of the designed vaccine candidate when bound to TLR3. The majority of residues exhibit low fluctuation, indicating restricted atomic movement and structural stability. Minor peaks represent flexible loop regions or surface-exposed residues, which is typical and does not compromise the overall complex stability. These observations confirm that the vaccine maintains a stable conformation when interacting with TLR3.

## Immune stimulation:

The C-ImmSim simulator was used to emulate the immune response to the pathogens [21]. The vaccine sequence was inputted in FASTA format, and all simulation parameters were at their default values [22]. Time steps were set as in to replicate repeated antigen exposures, as observed in typical endemic regions, thereby facilitating the investigation of clonal selection. The workflow of the entire methodology has been shown in Figure 1 - (see PDF).

## Results and Discussion:

Epitope-based vaccines offer a promising strategy for eliciting a targeted and effective immune response while minimizing interactions with non-relevant epitopes, thereby enhancing safety. Additionally, this approach allows for the precise selection and engineering of epitopes, optimizing potency and directing the immune system toward conserved antigenic regions. In light of these advantages, this study focuses on the in silico identification and design of a multi-epitope vaccine candidate targeting immunogenic proteins of *C. difficile*. A comprehensive subtractive proteomic analysis was conducted to identify a suitable vaccine target from *Clostridioides difficile*. Following a systematic screening of unique bacterial proteins, the ABC-type transport system, lantibiotic/multidrug-family ATP-binding protein from *Clostridioides difficile* strain 630 was selected based on its predicted virulence, antigenicity and potential immunogenicity. To design a multi-epitope vaccine, B-cell and T-cell epitopes predicted from the selected immunogenic proteins were linked using appropriate peptide linkers. Specialized spacer sequences were incorporated to enhance vaccine stability and efficacy. To minimize junctional immunogenicity and ensure a rational vaccine design, AAY and GPGPG linkers were used between epitopes. Additionally, an EAAAK linker, commonly employed in bifunctional proteins [23], was incorporated between the adjuvant sequence and fused epitopes to improve expression levels and bioactivity. The designed vaccine construct has a molecular weight of 6554.57 kilodaltons and exhibits favourable physicochemical properties. The theoretical isoelectric point (pI) of 9.82 indicates a basic nature, while the predicted instability index classifies it as stable. Furthermore, solubility predictions suggest that the recombinant protein maintains high thermal stability, reinforcing its suitability as a vaccine candidate.

Structural analysis revealed that the designed protein primarily consists of coil structures, contributing to approximately 60% of the total conformation. Further optimization of the vaccine candidate led to an improved three-dimensional (3D) structural model. Validation using a Ramachandran plot confirmed the accuracy of the model, with 89.6% of amino acid residues positioned in favoured regions and only 2.1% in disallowed regions. These findings support the structural reliability and potential immunogenic effectiveness of the designed multi-epitope vaccine. Further experiment was conducted on Protein - protein docking and this revealed that the residues interact favorably with TLR3. TLR3 is important in the enhancement of cross presentation of antigens and CD8+ T-cells activity. The designed chimeric vaccine was docked with TLR3 due to its critical role in innate immune recognition and activation of adaptive immunity. TLR3 is a pattern recognition receptor that detects viral and bacterial dsRNA, leading to the activation of antigen-presenting cells and the stimulation of pro-inflammatory cytokines, particularly interferon-gamma (IFN-γ) and interleukin-2 (IL-2). By targeting TLR3, the vaccine aims to enhance antigen presentation, promote a strong Th1 immune response and facilitate robust activation of cytotoxic T lymphocytes (CTLs). This interaction is crucial for ensuring the vaccine's efficacy in eliciting a protective immune response against *Clostridioides difficile*, making TLR3 a strategic docking target for evaluating the vaccine's immunogenic potential. Molecular docking of the human TL3 receptor PDB ID: 2AOZ with the vaccine was done using HDOCK protein-protein server the best 10 models were chosen and out of which model no 4 was selected as it had least binding energy for molecular dynamics simulation studies. The docking pose of TLR3-designed peptide vaccine is given in Figure 3 - (see PDF). Thus, in our molecular dynamics simulation it was found that the peptide vaccine and TLR3 complex had converged beyond 90 ns and final convergence was observed at the end of 100 ns. Figure 4 - (see PDF) depicts the evolution of the docked complex conformation over the 100 ns simulation. The RMSD Plots of the entire protein became stable with very minimal fluctuation of 0.1Å and the initial deviations which started from 1Å-2Å. The RMSD and RMSF plots are shown in Figure 5 - (see PDF) and Figure 6 - (see PDF) respectively. After the repeated immune provocations by the antigen, there seemed to be general upswings in the immune reactions. Immunological responses were characterized by the emergence of memory B and T cell profiles against the efficient component of the virus and individual B-cell memory spanning to several months. The Helper T cells were induced more actively. Moreover, IFN-γ and IL-2 responses elevated after the first injection, overtime it remained at the maximum level. The continuously high levels of IFN-γ, IL-2 cytokines in blood post vaccination suggest efficient immunoglobulin production, thus pointing to a good immunoglobulin response and relevant humoral immune response.

The current study focuses on the design of a multi-epitope vaccine against *Clostridioides difficile* using the ABC-type transport system protein, differs from the referenced studies [24-25] in several key aspects. Firstly, the current study specifically targets the ABC-type transport system protein, a novel candidate selected through subtractive proteomics for its high immunogenicity and pathogen specificity, whereas the referenced studies focus on different antigenic proteins, such as toxin A and toxin B or other surface proteins of *C. difficile*. Secondly, the vaccine construct integrates a unique combination of B-cell, CTL, and HTL epitopes with AAY and GPGPG linkers, coupled with a TLR3 adjuvant, designed to enhance innate and adaptive immune responses, which contrasts with the epitope selection and adjuvant strategies employed in the referenced works. The immune simulation using C-ImmSim predicts robust B- and T-cell responses with elevated IFN-γ and IL-2 levels, emphasizing a tailored prophylactic approach against antibiotic-resistant *C. difficile* strains, which is distinct from the therapeutic or broader immunological focus of the referenced studies.

## Conclusion:

A multi-epitope vaccine targeting *Clostridioides difficile* using the highly immunogenic ABC-type transport system protein and incorporating B-cell, CTL, and HTL epitopes with a TLR3 adjuvant was reported. The 66-amino-acid construct demonstrated strong antigenicity, structural stability, and high receptor-binding affinity, as confirmed through structural modelling, molecular docking, and molecular dynamics simulations. Predicted immune simulations revealed robust humoral and cellular responses, including elevated IFN-γ and IL-2 levels, highlighting its potential as a cost-effective and targeted prophylactic solution against antibiotic-resistant and recurrent *C. difficile* infections for further consideration.

## List of abbreviations:

*C. diff* - *Clostridioides difficile*;

CDI - Clostridioides Disease Infection;

TH - T Helper Cell;

CTL - Cytotoxic T Cell;

IFN-γ - Interferon Gamma;

TLR - Toll like Receptor
